# Unstimulated whole saliva flow for diagnosis of primary Sjögren’s syndrome: time to revisit the threshold?

**DOI:** 10.1186/s13075-020-2132-3

**Published:** 2020-02-24

**Authors:** Valentin Lacombe, Carole Lacout, Pierre Lozac’h, Alaa Ghali, Aline Gury, Christian Lavigne, Geoffrey Urbanski

**Affiliations:** Department of Internal Medicine, University Hospital, 4 rue Larrey, 49000 Angers, France

**Keywords:** Sjögren’s syndrome, Xerostomia, Saliva, Sex, Age groups, Classification

## Abstract

**Background:**

Unstimulated whole saliva (UWS) flow rate is one of the ACR/EULAR 2016 criteria for primary Sjögren’s syndrome (pSS). With a single threshold of ≤ 0.1 mL/min, UWS flow does not take into account the age- and sex-related physiological variations. Furthermore, it has a low sensitivity for the diagnosis of pSS (about 50%), contrary to the screening test for xerophthalmia, Schirmer’s test (sensitivity of about 70%). We aimed to identify UWS thresholds allowing better performances for a screening test for pSS comparable to Schirmer’s test, and considering age- and sex-related variations.

**Methods:**

A prospective cohort of 185 patients with oral and/or ocular dryness was classified into 3 groups: men, women < 50 (< 50 years old), and women ≥ 50 (≥ 50 years old). The diagnostic performances of UWS flow rate in these groups were compared in terms of sensitivity, specificity, positive and negative predictive values, and ROC curves. The identification of thresholds that optimize diagnostic performances was carried out using Youden’s index.

**Results:**

The diagnostic performances of UWS flow rate varied according to age and sex. UWS had poor diagnostic performances whatever the threshold in the women ≥ 50 group. The threshold of 0.2 mL/min had a sensitivity of ≥ 70% and a specificity of ≥ 50% in both men and women < 50 groups. In the whole population and compared to the current cutoff, a threshold of 0.2 mL/min increased sensitivity (+ 19.8%) and positive (+ 2.3%) and negative (+ 7.0%) predictive values, with a better specificity (65.2%) than Schirmer’s test.

**Conclusion:**

For objective assessment of xerostomia, raising the threshold of the UWS flow rate to 0.2 mL/min would optimize its screening performances for pSS.

## Background

Xerostomia is the main symptom of primary Sjögren’s syndrome (pSS). The objective assessment of xerostomia by unstimulated whole saliva (UWS) flow rate with a threshold of ≤ 0.1 mL/min is one of the ACR/EULAR 2016 criteria for pSS [1, 2]. In the absence of international consensus for the diagnosis of pSS, Vitali et al. have proposed the threshold of 0.1 mL/min in 1993, based on limited data from a study about 8 patients with pSS and 17 with secondary Sjögren’s syndrome [3, 4]. To our knowledge, this threshold has not been questioned in the following pSS classification criteria [1, 5, 6], and few studies were interested in its relevance.

Dryness of the mouth and/or eyes is the first symptom that gives rise to suspicion of pSS [2]. The objective assessment of these symptoms should have a high sensitivity level to screen patients who require more specific tests. In the ACR/EULAR 2016 score, the confirmation of pSS diagnosis is based on specific paraclinical tests: the presence of anti-SSA antibodies and/or significant lymphocytic infiltrate (focus score ≥ 1) on the minor salivary gland biopsy (MSGB). The choice of thresholds for ocular and salivary tests is therefore important. The salivary test threshold must also take into account the deterioration of saliva production with the progression of the pSS, meaning that patients at early stages may have higher UWS flows than those reported in cohorts [7].

Schirmer’s test with a threshold of ≤ 5 mm/5 min has a sensitivity of around 70% (42 to 90% according to studies) and a specificity of around 50% (from 34 to 76%) to establish the diagnosis of pSS (diagnosed according to different classification criteria between 2003 and 2016) [8–12]. Diagnostic performances of ocular and salivary tests should be comparable and have a high sensitivity, similar to that of Schirmer’s test in order to encourage clinicians to deepen investigations with more specific tests. However, the sensitivity of the UWS flow rate with a threshold of ≤ 0.1 mL/min showed 52% of sensitivity in a previous study [4]. Median UWS flows between 0.08 and 0.16 mL/min were observed in recent cohorts of pSS patients (diagnosed according to the two most recent international classification criteria), which confirmed the relatively low proportion of pSS patients having a UWS flow rate of ≤ 0.1 mL/min [13–16].

In a prospective cohort of 159 healthy subjects, Fenoll-Palomares et al. noted age- and sex-related variations in UWS flow: age < 44 years (*p* = 0.001) and male sex (*p = *0.02) were associated with a 2–3 times higher median UWS flow rates [17]. This sex-related difference in UWS flow has been previously observed [18], and lower UWS flow rates were also found among subjects above 60 years old compared to subjects aged between 18 and 40 in a meta-analysis (OR 0.55 [95% CI, 0.42–0.68], *p* < 0.001) [19]. Moreover, pSS often occurs around the menopause with a peak age between 40 and 55 years [20] while menopausal women have already physiologically reduced salivary flow. These physiological variations of UWS according to age and sex raise the question about the relevance of only single UWS threshold in criteria for pSS diagnosis for the whole population.

This study aimed at (i) testing the diagnostic performances for pSS of the UWS flow rate with a threshold of 0.1 mL/min realized in a prospective cohort of patients examined for oral and/or ocular dryness syndrome, (ii) testing whether the UWS flow performances vary according to age and sex, and (iii) identifying UWS thresholds having a level of sensitivity (≥ 70%) and specificity (≥ 50%) comparable to those of Schirmer’s test and to test their predictive values.

## Methods

### Ethics and statement for the study checklist

This study was approved by the ethics committee of Angers University Hospital (2014-5) and was conducted in compliance with the Declaration of Helsinki. This study applied the Strengthening the Reporting of Observational Studies in Epidemiology (STROBE) statement to observational studies.

### Selection of patients and composition of groups

We prospectively gathered the data of patients who were registered in the initial examination with oral and/or ocular dryness, evaluated by the same physician in an internal medicine department between January 2015 and June 2018. The initial reason for consultation could be directly dryness symptoms or diverse extraglandular symptoms leading to pSS suspicion. We used a standardized method to collect the following elements: subjective xerophthalmia and xerostomia, UWS flow rate, Schirmer’s test, MSGB, and anti-SSA and anti-SSB antibody testing. An ocular staining score (OSS) was given for patients presenting anti-SSA antibodies or a focus score of ≥ 1 in the MSGB (3 points according to the ACR/EULAR 2016 criteria) but negative results in Schirmer’s test or in the UWS flow rate.

Patients were classified into three groups: men, women < 50 (women younger than 50 years old), and women ≥ 50 (women aged 50 years old and above). The threshold age of 50 was chosen to approximate the menopausal status, 50 years corresponding to the mean age of menopause [21].

### Assessment of unstimulated whole saliva flow

The UWS flow measurement was carried out over a period of 15 min, as recommended [22]. The test was carried out between 9:30 am and 11:30 am after 3-h fasting and with no tobacco consumption since the day before. Patients were asked to swallow their saliva before the start of the test and then to spit into a container for 15 min. The volume of saliva was measured after decantation using a syringe calibrated at near to 0.1 mL.

### Diagnosis of primary Sjögren’s syndrome

The pSS diagnosis was made with a score ≥ 4 points according to the ACR/EULAR 2016 classification criteria: presence of lymphocytic sialadenitis with a focus score ≥ 1/4 mm^2^ on the MSGB (3 points), presence of anti-SSA antibodies (3 points), Schirmer’s test ≤ 5 mm/5 min in at least one eye (1 point), UWS flow rate ≤ 0.1 mL/min (1 point), and OSS ≥ 5 in at least one eye (1 point) [1]. We excluded patients who presented exclusion criteria according to the ACR/EULAR 2016 classification, and secondary Sjögren’s syndromes. Treatments causing iatrogenic oral and/or ocular dryness were identified [23, 24].

### Statistical analysis

The quantitative data are presented in medians and quartiles, compared using an ANOVA or the Kruskal-Wallis test according to distribution normality, which was evaluated using the d’Agostino-Pearson test. The qualitative data were presented as absolute values and as percentages and were compared using a chi-squared test. Diagnostic performances were evaluated by ROC curves with the calculation of the area under the curve (AUC) for continuous variables and by the sensitivity (Se), specificity (Sp), positive predictive values (PPV), and negative predictive values (NPV) for qualitative variables. To determine the UWS flow thresholds of interest among all the values by an interval of 0.1 mL/15 min, we used ROC curves and Youden’s index, defined by Se+Sp-1 (included between − 1 in cases of zero sensitivity and specificity and 1 in cases of 100% sensitivity and specificity), in order to appreciate the best pairing of sensitivity/specificity. The strength of association between the tested threshold and the pSS diagnosis was verified by Yule’s *Q* coefficient (none if *Q* = 0, insignificant if *Q* = (0.01–0.09), minor if *Q* = (0.10–0.29), moderate if *Q* = (0.30–0.49), strong if *Q* = (0.50–0.69), very strong if *Q* = (0.70–1)). Sensitivity analyses were performed in the men group, to verify if the best threshold found in whole men was influenced by age. Sensitivity analyses were also performed according to the results of the MSGB and the anti-SSA assay, to verify if the best threshold found in the whole population was influenced by these results. A linear regression model was performed to assess the impact of age on the UWS flow rate with UWS flow rate as dependent variable and age as explicative one.

In order to identify interesting UWS flow thresholds, we first used the current pSS ACR/EULAR 2016 classification criteria with UWS flow ≤ 0.1 mL/min to confirm pSS diagnosis. Secondly, to test the new thresholds that we suggested, we adapted the score from the ACR/EULAR 2016 classification criteria (adapted score from ACR/EULAR 2016 criteria) by modifying the level of the UWS flow rate cutoff to give 1 point: a patient presenting a UWS flow lower than or equal to the tested threshold obtained 1 point on the adapted ACR/EULAR 2016 criteria score.

The alpha risk was 5%. The indices were presented with a confidence interval of 95%. The analyses were carried out using the GraphPad Prism v6.01 software (GraphPad Software, Inc., La Jolla, CA 92037, USA).

## Results

### Description of the three groups

The general characteristics of the 3 groups are summarized in Table 1. The cohort included 185 patients with a median age of 57 [48–67] years, with 38 (20.5%) patients in the men group, 42 (22.7%) in the women < 50 group, and 105 (56.8%) in the women ≥ 50 group. pSS was diagnosed in 93 (50.3%) patients and was more frequent in the women ≥ 50 group (*p* = 0.048).

**Table 1:** Characteristics of the three groups

	Men	Women < 50	Women ≥ 50	*p* value
Number of patients	38	42	105	
Age (years)	57 [46–68]	37 [30–46]	64 [55–68]	< 0.0001
pSS	16 (42.1%)	16 (38.1%)	61 (58.1%)	0.048
Treatments inducing oral and/or ocular dryness	5 (13.2%)	10 (23.8%)	28 (26.7%)	0.24
Subjective DES	28 (73.7%)	40 (95.2%)	95 (90.5%)	< 0.01
Schirmer^a^ (mm/5 min)	1 [0–3]	5 [1–17.3]	1 [0–4]	< 0.001
Schirmer ≤ 5 mm/5 min	33 (86.8%)	25 (59.5%)	90 (85.7%)	< 0.001
OSS realization	10 (26.3%)	10 (23.8%)	29 (27.6%)	0.69
OSS ≥ 5 (if examination was realized)^b^	1/10 (10.0%)	2/10 (20.0%)	8/29 (27.6%)	0.51
Subjective DMS	30 (78.9%)	37 (88.1%)	99 (94.3%)	0.026
UWS flow (mL/min)	0.27 [0.08–0.42]	0.21 [0.09–0.46]	0.20 [0.07–0.33]	0.25
UWS flow rate ≤ 0.1 mL/min	11 (28.9%)	13 (31.0%)	40 (38.1%)	0.51
Anti-SSA antibodies	4 (10.5%)	9 (21.4%)	22 (21.0%)	0.35
Anti-SSB antibodies	5 (13.2%)	6 (14.3%)	16 (15.2%)	0.95
ANA > 1/200	12 (31.6%)	12 (28.6%)	32 (30.5%)	0.96
Focus score ≥ 1 on MSGB	15 (39.5%)	17 (40.5%)	61 (58.1%)	0.051

*ANA* anti-nuclear antibodies, *MSGB* minor salivary gland biopsy, *UWS* unstimulated whole saliva, *OSS* ocular staining score, *pSS* primary Sjögren’s syndrome, *DMS* dry mouth syndrome, *DES* dry eye syndrome

^a^Lowest value of both eyes

^b^The percentage given corresponds to the proportion of patients with an OSS ≥ 5 among those who underwent this examination

The median UWS flow did not differ significantly between the three groups, but varied according to the results of the MSGB: 0.27 mL/min [0.10–0.45] in cases of a focus score < 1 against 0.13 mL/min [0.07–0.32] in cases of a focus score ≥ 1 (*p* <  0.0001) among the whole population. In patients with pSS, the median UWS flow did not differ significantly in terms of MSGB results (0.13 mL/min [0.07–0.32] if the focus score is < 1 against 0.12 mL/min [0.03–0.27] if the focus score is ≥ 1, *p* = 0.54) and in terms of anti-SSA antibody testing (0.13 mL/min [0.07–0.32] in the absence of anti-SSA antibodies against 0.12 mL/min [0.06–0.29] in the presence of anti-SSA antibodies, *p* = 0.47). UWS flow did not differ according to the results of Schirmer’s test (*p* = 0.26) or OSS (*p* = 0.27). Sensitivity and specificity of subjective xerostomia were 91% and 9%, respectively, to predict a UWS ≤ 0.1 mL/min.

### UWS flow rate according to age and sex

**Fig. 1:**
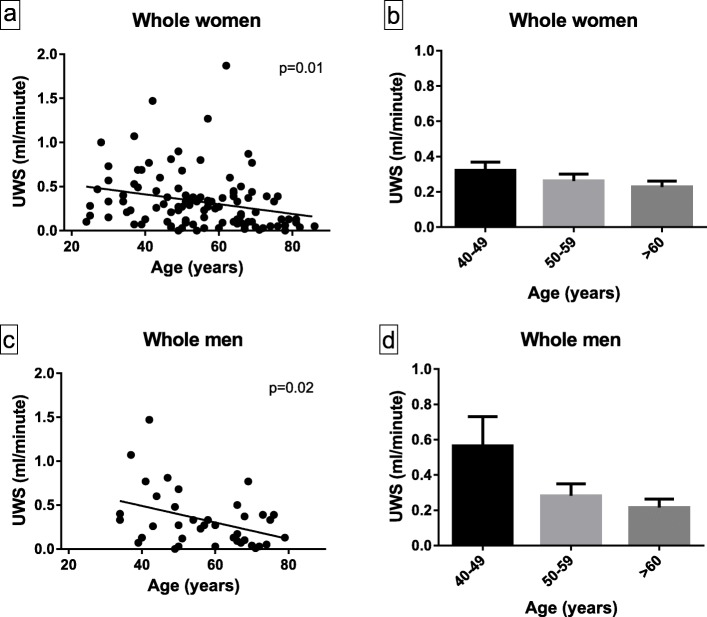
Evolution of unstimulated whole saliva (UWS) flow rate with age, in women (**a**, **b**) and in men (**c**, **d**)

In women and men, a significant decrease of UWS flow rate was observed in the whole population with age (Fig. 1). In women as in men, a decrease of UWS flow rate was observed at 50 years old. Treatments with anticholinergic effect were not more frequent in women ≥ 50 years versus women < 50 years (26.7% vs 23.8%, *p* = 0.63) and in men ≥ 50 years versus women < 50 years (16.7% vs 11.5%, *p* = 0.64).

### Diagnostic performances

#### Diagnostic performances of items from current criteria of ACR/EULAR 2016 for pSS

The diagnostic performances of items from the ACR/EULAR 2016 score are summarized in Table 2. Among the 185 patients, Schirmer’s test with a threshold of ≤ 5 mm/5 min had a sensitivity of 87.1% [95% CI, 78.6–93.2] and a specificity of 30.4% [95% CI, 21.3–40.9]. UWS flow rate with a threshold of ≤ 0.1 mL/min had a sensitivity of 43.0% [95% CI, 32.8–53.7] and a specificity of 73.9% [95% CI, 63.7–82.5] for the diagnosis of pSS.

**Table 2:** Diagnostic performances of criteria in the ACR/EULAR 2016 score

	Schirmer ≤ 5 mm/5 min	UWS ≤ 0.1 mL/min	Anti-SSA antibodies	Focus score ≥ 1/4 mm^2^
Whole population (*n* = 185)				
Se (%)	89.2 [81.1–94.7]	43.0 [32.8–53.7]	37.6 [27.8–48.3]	91.4 [83.8–96.2]
Sp (%)	29.3 [20.3–39.8]	73.9 [63.7–82.5]	100 [96.1–100]	91.3 [83.6–96.2]
PPV (%)	56.1 [47.7–64.2]	62.5 [49.5–74.3]	100 [90.0–100]	91.4 [83.8–96.2]
NPV (%)	73.0 [55.9–86.2]	56.2 [46.9–65.2]	61.3 [53.0–69.2]	91.3 [83.6–96.2]
Men (*n* = 38)				
Se (%)	100 [79.4–100]	56.3 [29.9–80.2]	25.0 [7.3–52.4]	87.5 [61.7–98.4]
Sp (%)	22.7 [7.8–45.4]	90.9 [70.8–98.9]	100 [84.6–100]	95.5 [77.2–99.9]
PPV (%)	48.5 [30.8–66.5]	81.8 [48.2–97.7]	100 [39.8–100]	93.3 [68.1–99.8]
NPV (%)	100 [47.8–100]	74.1 [53.7–88.9]	64.7 [46.5–80.3]	91.3 [72.0–98.9]
Women < 50 (*n* = 42)				
Se (%)	68.8 [41.3–89.0]	50.0 [54.7–75.3]	56.3 [29.9–80.2]	93.8 [69.8–99.8]
Sp (%)	46.2 [26.6–66.6]	80.8 [60.6–93.4]	100 [86.8–100]	92.3 [74.9–99.1]
PPV (%)	44.0 [24.4–65.1]	61.5 [31.6–86.1]	100 [66.4–100]	88.2 [63.6–98.5]
NPV (%)	70.6 [44.0–89.7]	72.4 [52.8–87.3]	78.8 [61.1–91.0]	96.0 [79.6–99.9]
Women ≥ 50 (*n* = 105)				
Se (%)	91.8 [81.9–97.3]	37.7 [25.6–51.0]	36.1 [24.2–49.4]	91.8 [81.9–97.3]
Sp (%)	22.7 [11.5–37.8]	61.4 [45.5–75.6]	100 [92.0–100]	88.6 [75.4–96.2]
PPV (%)	62.2 [51.4–72.2]	57.5 [40.9–73.0]	100 [84.6–100]	91.8 [81.9–97.3]
NPV (%)	66.7 [38.4–88.2]	41.5 [29.4–54.4]	53.0 [41.7–64.1]	88.6 [75.4–96.2]

*UWS* unstimulated whole saliva flow rate, *Se* sensitivity, *Sp* specificity, *PPV* positive predictive value, *NPV* negative predictive value

#### Analysis of UWS flow as a continuous variable (ROC curves) according to sex and age (Fig. 2)

The performances of UWS flow for pSS diagnosis were only significant in the men and women < 50 groups with AUCs of 0.81 [95% CI, 0.68–0.96] (*p* = 0.001) and 0.78 [95% CI, 0.62–0.93] (*p* = 0.003), respectively. The AUC of the women ≥ 50 group was 0.59 [95% CI, 0.47–0.70] (*p* = 0.12).

**Fig. 2:**
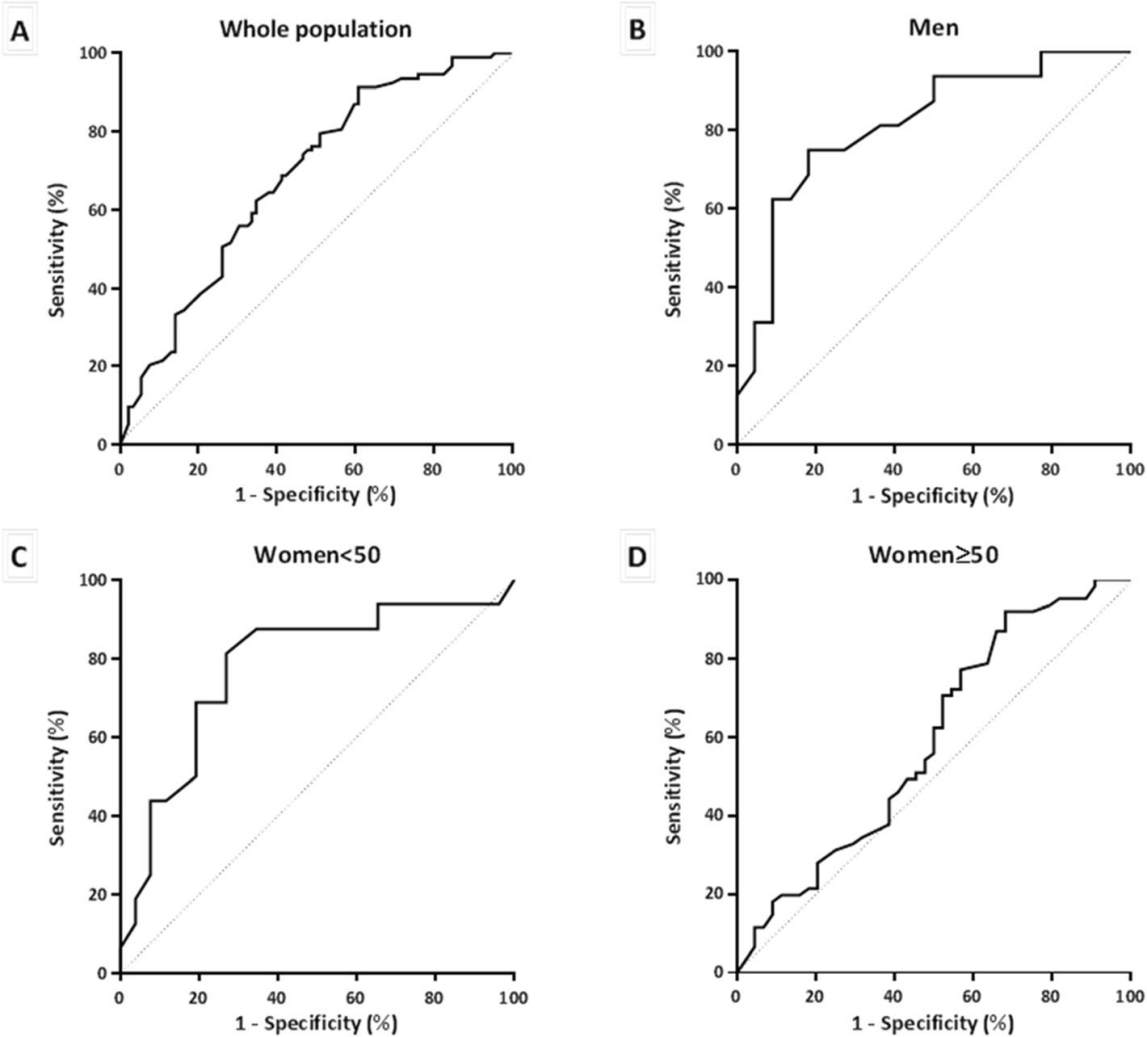
ROC curves representing the diagnostic value of UWS flow in primary Sjögren’s syndrome in the whole population and in each group

#### Identification of best UWS flow thresholds according to age and sex and evaluation of their diagnostic performances (Table 3)

**Fig. 3:**
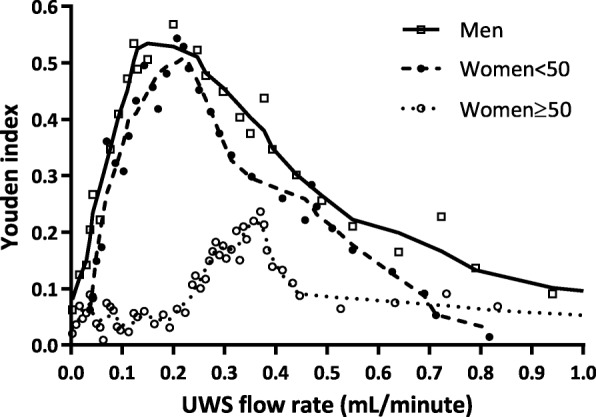
Youden’s index according to the saliva flow rate in each group. UWS, unstimulated whole saliva

The UWS flow thresholds with the best pairing of Se/Sp were 0.2 mL/min in both the men group (Youden’s index 0.57) and the women < 50 group (Youden’s index 0.49) and 0.33 mL/min in the women ≥ 50 group (Youden’s index 0.21) (Fig. 3). With a threshold of 0.2 mL/min, Yule’s *Q* index was very strong in the men and women < 50 groups (0.86 and 0.83, respectively) and minor in the women > 50 group (0.13). Diagnostic performances of UWS with a threshold of 0.2 mL/min in the three groups are presented in Table 3.

**Table 3:** Diagnostic performances of unstimulated whole saliva flow rate with the adapted score from ACR/EULAR 2016 criteria for pSS according to the threshold

	Whole population		Men		Women < 50		Women > 50	
UWS (mL/min)	≤ 0.1	≤ 0.2	≤ 0.1	≤ 0.2	≤ 0.1	≤ 0.2	≤ 0.1	≤ 0.2
Diagnostic performances of UWS flow rate with the adapted ACR/EULAR 2016 score								
Se	43.0 [32.8–53.7]	62.8 [52.2–72.5]	56.3 [29.9–80.2]	75.0 [47.6–92.7]	50.0 [24.7–75.3]	82.4 [56.6–96.2]	37.7 [25.6–51.0]	54.1 [40.8–66.9]
Sp	73.9 [63.7–82.5]	65.2 [54.6–74.9]	90.9 [70.8–98.9]	81.8 [59.7–94.8]	80.8 [60.6–93.4]	76.0 [54.9–90.6]	61.4 [45.5–75.6]	52.3 [36.7–67.5]
YI	0.17	0.28	0.47	0.57	0.31	0.58	− 0.01	0.06
PPV	62.5 [49.5–74.3]	64.8 [54.1–74.6]	81.8 [48.2–97.7]	75.0 [47.6–92.7]	61.5 [31.6–86.1]	70.0 [45.7–88.1]	57.5 [40.9–73.0]	61.1 [46.9–74.1]
NPV	56.2 [46.9–65.2]	63.2 [52.6–72.8]	74.1 [53.7–88.9]	81.8 [59.7–94.8]	72.4 [52.8–87.3]	86.4 [65.1–97.1]	41.5 [29.4–54.4]	45.1 [31.1–59.7]

The adapted ACR/EULAR 2016 score was adapted from the ACR/EULAR 2016 classification criteria by modifying the level of the UWS flow rate cutoff to give 1 point for this item: a patient presenting a UWS flow lower than or equal to our tested threshold obtained 1 point on the adapted score

*UWS* unstimulated whole saliva, *Se* sensitivity, *Sp* specificity, *PPV* positive predictive value, *NPV* negative predictive value, *YI* Youden’s index

Sensitivity analyses have been performed in the men group. A threshold age of 50 years was chosen due to the abrupt decrease of UWS observed in our cohort, as previously detailed. After subgrouping men into two subgroups, men < 50 (men < 50 years old) and men ≥ 50 (men of 50 years old or older), the threshold of 0.2 mL/min appeared as the best threshold in both subgroups, as observed in the whole men population. Indeed, this threshold of 0.2 mL/min was associated with Se of 100% and Sp of 100% (Youden’s index 1.0) in the men < 50 subgroup (compared to 67% and 100%, respectively, with the actual threshold of 0.1 mL/min, Youden’s index 0.67) and Se of 69% and Sp of 69% (Youden’s index 0.38) in the men ≥ 50 subgroup (compared to 46% and 85% with the current threshold of 0.1 mL/min, Youden’s index 0.31).

Sensitivity analyses have been performed according to the results of the MSGB and the anti-SSA antibodies assay. In patients without a focus score of ≥ 1, the threshold of 0.1 mL/min was associated with a sensitivity of 25%, a specificity of 77%, and Youden’s index of 0.02, whereas the threshold of 0.2 mL/min was associated with a sensitivity of 50%, a specificity of 63%, and Youden’s index of 0.13 for the diagnosis of pSS. In patients without anti-SSA antibodies, the threshold of 0.1 mL/min was associated with a sensitivity of 36%, a specificity of 79%, and Youden’s index of 0.15, whereas the threshold of 0.2 mL/min was associated with a sensitivity of 62%, a specificity of 65%, and Youden’s index of 0.27.

The UWS flow threshold of 0.2 mL/min in the whole population had a Se of 62.8% [95% CI, 52.2–72.5], a Sp of 65.2% [95% CI, 54.6–74.9], a PPV of 64.8% [95% CI, 54.1–74.6], and a NPV of 63.2% [95% CI, 52.6–72.8].

#### Diagnostic performances of UWS flow threshold of 0.2 mL/min according to the adapted score from ACR/EULAR 2016 classification criteria (Table 3)

The comparison of the diagnostic performances of the UWS flow rate between the thresholds of 0.1 mL/min in the current score from ACR/EULAR 2016 criteria and of 0.2 mL/min in the adapted ACR/EULAR 2016 score showed an increase in sensitivity of + 18.7% in the men group, + 32.4% in the women< 50 group, and + 16.4% in the women ≥ 50 group with a threshold of 0.2 mL/min. The specificity reduced by − 9.1% in the men group, − 4.8% in the women < 50 group, and − 9.1% in the women ≥ 50 group with the threshold of 0.2 mL/min. In the whole population, both PPV and NPV increase with a threshold of 0.2 mL/min: + 2.3% and + 7.0% compared with the current threshold.

## Discussion

pSS is a systemic disease that is mainly characterized by glandular involvement resulting in significant morbidity [25–28]. pSS is often suspected when patients report persistent oral and/or ocular dryness. Screening for pSS is realized by easy tests: Schirmer’s test and UWS flow rate. The diagnostic performances of UWS flow in pSS have already been evaluated, but to our knowledge, no study has reconsidered its threshold according to age and sex [4]. The UWS threshold of 0.1 mL/min was chosen in 1993 to define a homogeneous group of patients with pSS diagnosis in order to harmonize clinical practices and researches, whereas there were no consensual classification criteria at this time [3]. Since then, the context has changed: we now have an international consensus for classification criteria, and Sjögren’s syndrome is better recognized. Nowadays, UWS flow and Schirmer’s tests aim to screen patients for pSS diagnosis and need to be associated with a sufficient level of sensitivity to allow an objective assessment of oral and/or ocular dryness before a diagnostic confirmation with specific immunological or histological tests [29]. This does not concern patients presenting with a severe extraglandular involvement, in which MSGB and immunological assays should be performed even without subjective or objective dryness. In line with previous studies, we found that Schirmer’s test ≤ 5 mm/5 min had a high sensitivity (89.2%), while a UWS flow rate of ≤ 0.1 mL/min was substantially less sensitive (43.0%) [8–12]. So, we aimed at identifying UWS flow thresholds that can be adapted to age- and sex-related variations observed in healthy population to optimize the diagnostic performances of this frequently used test.

The very low specificity of subjective xerostomia for assessing objective buccal dryness (9% in our study) confirmed the need to use objective tests. This is in accordance with the most recent international classification criteria, ACR/EULAR 2016, where subjective xerophthalmia and xerostomia are no longer part of the items of scoring [1]. We also confirmed that UWS flow rate decreases with age in both women and men [19]. One could think that treatments with anticholinergic effects (especially antihypertensive treatments) may explain the decrease of UWS in older people, but these treatments were not more frequent in subjects ≥ 50 years than in subjects < 50 years in our cohort. This association between age and UWS flow rate confirmed the need to study UWS threshold according to age.

In the women ≥ 50 group, the UWS flow rate showed poor diagnostic performances regardless of the tested thresholds. This could be explained by the physiological reduction of UWS flow in these patients due to the submandibular and sublingual gland atrophy and fibrosis [19], which limits the specificity of the UWS flow rate. We were unable to find a cutoff that is sufficiently sensitive without an important loss of specificity for women aged 50 years and older. In contrast, in both men and women < 50 groups, the UWS flow cutoff value of 0.2 mL/min seems to be interesting and allows obtaining the best performances.

In the women ≥ 50 group, the threshold of 0.2 mL/min makes it possible to increase sensitivity without loss of specificity, while a threshold of 0.33 mL/min significantly reduced the specificity (− 27.3% compared to the threshold of 0.1 mL/min). Due to the epidemiology of Sjögren’s syndrome, screening for pSS will be easier in women ≥ 50 years than in men and younger women. So, it is important to favor a threshold that is adapted for younger women and men who are at risk of underdiagnosis.

Considering the whole population and compared with the current threshold of UWS flow, a threshold of 0.2 mL/min increased the sensitivity of + 19.8%, while keeping a much better specificity (65.2% [95% CI, 54.6–74.9]) than Schirmer’s test (29.3% [95% CI, 20.3–39.8]) in our study. Moreover, the threshold of 0.2 mL/min allowed to improve both PPV and NPV (+ 2.3% and + 7.0%, respectively) in the whole population.

To test oral dryness, we measured the unstimulated whole saliva flow, which is recommended in the ACR/EULAR 2016 classification, due to its good reproducibility [1, 22]. The continuous unstimulated salivary secretion has homeostatic proprieties, and the UWS flow is negatively correlated with the number of dental caries [30]. Compared to stimulated salivary flow, the UWS test is simple to administer and has a good reproducibility and a better correlation with the clinical impact of xerostomia. However, and contrary to the stimulated salivary flow that varies little with age, the unstimulated salivary flow is reduced by 40 to 70% between the age of 20 and 80 years [18, 31]. This is why it is of great importance to study its diagnostic performances according to age.

In our study, all patients reported at least one symptom that could be related to Sjögren’s syndrome, allowing the use of ACR/EULAR 2016 classification criteria. Due to the fact that these criteria are based on objective clinical and paraclinical items, the diagnosis of pSS was not subject to interpretation. Both Schirmer’s test and UWS flow rates were carried out for all patients, regardless of whether or not they reported subjective oral dryness, to avoid any possible underestimation of dryness by some patients. In fact, it has been shown that oral dryness is perceived when the UWS flow is reduced by 40 to 50%, without the need to reach such a low flow level of 0.1 mL/min [32].

We used the international classification criteria to define pSS diagnosis, even if their diagnostic performances could be contested [33]. It is not excluded that some patients were wrongly diagnosed with pSS on the basis of focus score ≥ 1 on the MSGB together with reduced UWS flow due to another reason like age. Indeed, lymphocytic foci with focus score ≥ 1 may be observed in healthy subjects [34]. These data highlighted that it is of interest to consider conditions inducing dryness like age to define the UWS threshold. Moreover, some studies reported differences in patients with and without anti-SSA antibodies, with more extraglandular involvement in patients with anti-SSA antibodies, for example, renal involvement [35]. This may lead to questioning the pSS diagnosis in case of the absence of anti-SSA antibodies. Nevertheless, the expression of pSS in patients without anti-SSA antibodies is not limited to dryness symptoms, some studies reporting more peripheral nervous system involvement [36], and notably more small fiber neuropathy [37]. In our study, the UWS threshold of 0.2 mL/min was associated with better diagnosis performances than the actual threshold independently of the MSGB and anti-SSA antibodies results. The increase of sensitivity and decrease of specificity were similar in both subgroups of patients (Se + 25% and Sp − 14% in case of focus score < 1, Se + 26% and Sp − 14% in case of absence of anti-SSA antibodies).

The choice of the most suitable threshold to distinguish between patients with or without pSS has been determined from our cohort. Thus, our results need to be validated by a further prospective study.

## Conclusions

The diagnostic performances of UWS flow rate to establish a pSS diagnosis varied according to age and sex. In men and women younger than 50 years, a UWS flow threshold of 0.2 mL/min enabled better diagnostic performances than the current threshold of 0.1 mL/min. In women aged 50 years and older, the physiological reduction of salivary secretion considerably limited its specificity, but increasing the threshold of 0.2 mL/min appeared to increase the sensitivity for similar overall performances in this population. In the whole population and compared to the cutoff of 0.1 mL/min, a threshold of 0.2 mL/min increases sensitivity (+ 19.8%), PPV (+ 2.3%), and NPV (+ 7.0%), while keeping a better specificity than Schirmer’s test in our study. Thus, raising the threshold of the UWS flow rate to 0.2 mL/min in the ACR/EULAR 2016 score would allow a better selection of patients to explore with MSGB and anti-SSA antibody testing.

## Data Availability

All of the data supporting the conclusions of this article are included within the article.

## Abbreviations

MSGB: Minor salivary gland biopsy
pSS: Primary Sjögren’s syndrome
UWS: Unstimulated whole saliva

## References

[1] Shiboski CH, Shiboski SC, Seror R, Criswell LA, Labetoulle M, Lietman TM, et al. 2016 American College of Rheumatology/European League Against Rheumatism Classification Criteria for Primary Sjögren’s Syndrome: a consensus and data-driven methodology involving three international patient cohorts. Arthritis Rheumatol. 2017;69:35–45. 10.1002/art.39859 PMC5650478 27785888

[2] Mariette X, Criswell LA. Primary Sjögren’s syndrome. Solomon CG, editor. N Engl J Med 2018;378:931–939. 10.1056/NEJMcp1702514 29514034

[3] Vitali C, Bombardieri S, Moutsopoulos HM, Balestrieri G, Bencivelli W, Bernstein RM (1993). Preliminary criteria for the classification of Sjögren’s syndrome. Results of a prospective concerted action supported by the European community. Arthritis Rheum.

[4] Speight PM, Kaul A, Melsom RD (1992). Measurement of whole unstimulated salivary flow in the diagnosis of Sjögren’s syndrome. Ann Rheum Dis.

[5] Vitali C, Bombardieri S, Jonsson R, Moutsopoulos HM, Alexander EL, Carsons SE (2002). Classification criteria for Sjögren’s syndrome: a revised version of the European criteria proposed by the American-European Consensus Group. Ann Rheum Dis.

[6] Shiboski SC, Shiboski CH, Criswell LA, Baer AN, Challacombe S, Lanfranchi H (2012). American College of Rheumatology classification criteria for Sjögren’s syndrome: a data-driven, expert consensus approach in the Sjögren’s International Collaborative Clinical Alliance cohort. Arthritis Care Res.

[7] Azuma N, Katada Y, Sano H. Deterioration in saliva quality in patients with Sjögren’s syndrome: impact of decrease in salivary epidermal growth factor on the severity of intraoral manifestations. Inflamm Regen. 2018;38:6. 10.1186/s41232-018-0062-0 PMC5890343 29657585

[8] Vissink A, Kalk WWI, Mansour K, Spijkervet FKL, Bootsma H, Roodenburg JLN (2003). Comparison of lacrimal and salivary gland involvement in Sjögren’s syndrome. Arch Otolaryngol Neck Surg.

[9] Versura P, Frigato M, Mulé R, Malavolta N, Campos EC (2006). A proposal of new ocular items in Sjögren’s syndrome classification criteria. Clin Exp Rheumatol.

[10] Versura P, Frigato M, Cellini M, Mulè R, Malavolta N, Campos EC (2007). Diagnostic performance of tear function tests in Sjogren’s syndrome patients. Eye..

[11] Alves M, Reinach PS, Paula JS, Vellasco e Cruz AA, Bachette L, Faustino J, et al. Comparison of diagnostic tests in distinct well-defined conditions related to dry eye disease. Wedrich A, editor. PLoS One. 2014;9:e97921. 10.1371/journal.pone.0097921 PMC4029783 24848115

[12] Kim M, Kim HS, Na K-S (2017). Correlation between tear osmolarity and other ocular surface parameters in primary Sjögren’s syndrome. Korean J Ophthalmol.

[13] Çankaya H, Alpöz E, Karabulut G, Güneri P, Boyacıoglu H, Kabasakal Y (2010). Effects of hydroxychloroquine on salivary flow rates and oral complaints of Sjögren patients: a prospective sample study. Oral Surg Oral Med Oral Pathol Oral Radiol Endodontology.

[14] Baldini C, Luciano N, Tarantini G, Pascale R, Sernissi F, Mosca M, et al. Salivary gland ultrasonography: a highly specific tool for the early diagnosis of primary Sjögren’s syndrome. Arthritis Res Ther. 2015;17:146. 10.1186/s13075-015-0657-7 PMC4461980 26022533

[15] Bowman SJ, Everett CC, O’Dwyer JL, Emery P, Pitzalis C, Ng W-F, et al. Randomized controlled trial of rituximab and cost-effectiveness analysis in treating fatigue and oral dryness in primary Sjögren’s syndrome. Arthritis Rheumatol 2017;69:1440–1450. 10.1002/art.40093 28296257

[16] Fidelix T, Czapkowski A, Azjen S, Andriolo A, Neto PH, Trevisani V (2018). Low-level laser therapy for xerostomia in primary Sjögren’s syndrome: a randomized trial. Clin Rheumatol.

[17] Fenoll-Palomares C, Muñoz Montagud JV, Sanchiz V, Herreros B, Hernández V, Mínguez M (2004). Unstimulated salivary flow rate, pH and buffer capacity of saliva in healthy volunteers. Rev Espanola Enfermedades Dig Organo Of Soc Espanola Patol Dig.

[18] Percival RS, Challacombe S, Marsh PD (1994). Flow rates of resting whole and stimulated parotid saliva in relation to age and gender. J Dent Res.

[19] Affoo RH, Foley N, Garrick R, Siqueira WL, Martin RE (2015). Meta-analysis of salivary flow rates in young and older adults. J Am Geriatr Soc.

[20] Vivino FB (2017). Sjogren’s syndrome: clinical aspects. Clin Immunol.

[21] Pelosi E, Simonsick E, Forabosco A, Garcia-Ortiz JE, Schlessinger D. Dynamics of the ovarian reserve and impact of genetic and epidemiological factors on age of menopause. Biol Reprod. 2015;92:130. 10.1095/biolreprod.114.127381 PMC4645983 25904009

[22] Navazesh M, Kumar SKS (2008). Measuring salivary flow. J Am Dent Assoc.

[23] Baer AN, Walitt B (2017). Sjögren syndrome and other causes of sicca in older adults. Clin Geriatr Med.

[24] Wolff A, Joshi RK, Ekström J, Aframian D, Pedersen AML, Proctor G (2017). A guide to medications inducing salivary gland dysfunction, xerostomia, and subjective sialorrhea: a systematic review sponsored by the world workshop on oral medicine VI. Drugs RD.

[25] González S, Sung H, Sepúlveda D, González M, Molina C (2014). Oral manifestations and their treatment in Sjögren’s syndrome. Oral Dis.

[26] Cartee DL, Maker S, Dalonges D, Manski MC (2015). Sjögren’s syndrome: oral manifestations and treatment, a dental perspective. J Dent Hyg JDH.

[27] Holdgate N, St. Clair EW (2016). Recent advances in primary Sjogren’s syndrome. F1000Res.

[28] Zero DT, Brennan MT, Daniels TE, Papas A, Stewart C, Pinto A (2016). Clinical practice guidelines for oral management of Sjögren disease. J Am Dent Assoc..

[29] Liapi A, Horisberger A, François S, Ribi C (2016). Sjögren’s syndrome: when to suspect and how to confirm?. Rev Med Suisse.

[30] Daniels TE (2012). Do we need new diagnostic criteria for Sjögren’s syndrome?. Presse Med.

[31] Ben-Aryeh H, Miron D, Szargel R, Gutman D (1984). Clinical science whole-saliva secretion rates in old and young healthy subjects. J Dent Res.

[32] Dawes C (1987). Physiological factors affecting salivary flow rate, oral sugar clearance, and the sensation of dry mouth in man. J Dent Res.

[33] Le Goff M, Cornec D, Jousse-Joulin S, Guellec D, Costa S, Marhadour T, et al. Comparison of 2002 AECG and 2016 ACR/EULAR classification criteria and added value of salivary gland ultrasonography in a patient cohort with suspected primary Sjögren’s syndrome. Arthritis Res Ther. 2017;19:269. 10.1186/s13075-017-1475-x PMC5717850 29208023

[34] Radfar L, Kleiner DE, Fox PC, Pillemer SR (2002). Prevalence and clinical significance of lymphocytic foci in minor salivary glands of healthy volunteers. Arthritis Rheum.

[35] Luo J, Xu S, Lv Y, Huang X, Zhang H, Zhu X (2019). Clinical features and potential relevant factors of renal involvement in primary Sjögren’s syndrome. Int J Rheum Dis.

[36] Park Y, Lee J, Koh JH, Sung Y-K, Lee S-S, Choe JY (2019). Distinct clinical characteristics of anti-Ro/SSA-negative primary Sjögren’s syndrome: data from a nationwide cohort for Sjögren’s syndrome in Korea. Clin Exp Rheumatol.

[37] Birnbaum J, Lalji A, Saed A, Baer AN (2019). Biopsy-proven small-fiber neuropathy in primary Sjögren’s syndrome: neuropathic pain characteristics, autoantibody findings, and histopathologic features. Arthritis Care Res.

